# Clinical outcomes of internal fixation orthopaedic surgery in humanitarian settings: a retrospective cohort study at the Médecins Sans Frontières (MSF) trauma centre in Aden, Yemen

**DOI:** 10.1007/s00264-025-06616-y

**Published:** 2025-08-13

**Authors:** Rami Malaeb, Taha Hussain, Fares Ayyash, Abdulsalam Abdullah, Hameed S. Ahmed, Khaled Abdulrahman, Adel Al Haj, Hesham Bin Shahna, Evgenia Zelikova, Ibrahim Hassanin, Elisabeth Poulet, Patrick Herard, Rasheed Fakhri

**Affiliations:** 1 Epicentre, Paris, France; 2 Médecins Sans Frontières, Paris, France

**Keywords:** Internal fixation, Orthopedic surgery, Humanitarian settings, Bone union

## Abstract

**Purpose:**

The Aden Trauma Centre in Yemen, supported by Médecins Sans Frontières (MSF), introduced internal fixation (IF) procedures to address the high burden of fractures as a result of road traffic accidents and conflict-related injuries. This study aimed to describe the clinical characteristics of patients undergoing IF, evaluate their complication and healing outcomes, and explore factors influencing postoperative results.

**Methods:**

A retrospective cohort design was employed, including all patients who underwent internal fixation—using SIGN nails or plates/screws—between January and December 2022. Demographic information, fracture characteristics, surgical techniques, and postoperative outcomes were analyzed. Cox proportional hazards models were used to identify key predictors of complications and bone healing.

**Results:**

A total of 177 patients (208 fractures) were included. The overall complication rate was 14.4%. Open fractures and comorbidities were significant predictors of complications, while type of implant (SIGN nail vs. plate/screws) did not affect complication risk. Around three-quarters of fractures achieved radiographic healing at a median of five to six months. Infection and other complications emerged as major risk factors for delayed or impaired union. About a quarter of patients defaulted from care, potentially underestimating late complications and nonunion rates.

**Conclusion:**

Findings indicate that IF is feasible and effective in this high-need, low-resource context, demonstrating complication rates in line with global estimates. Open fractures, comorbidities, and limited follow-up infrastructure remain the main challenges to optimizing outcomes in such contexts.

**Supplementary Information:**

The online version contains supplementary material available at 10.1007/s00264-025-06616-y.

## Introduction

Trauma-related injuries are a leading cause of global mortality and disability, accounting for approximately 4.4 million deaths and 50 million disabilities annually. The burden is heaviest in low- and middle-income countries (LMICs), where inadequate infrastructure, personnel shortages, and operational constraints limit access to trauma care and surgery. An estimated two million deaths could be prevented annually with adequate trauma services [1, 2]. Addressing this burden requires targeted interventions, capacity building, and international collaboration to strengthen healthcare systems [3]. Armed conflicts and natural disasters further strain fragile infrastructures [4, 5]. Médecins Sans Frontières (MSF) plays a vital role in bridging trauma care gaps in these contexts, conducting over 125,000 surgical interventions globally in 2023, with trauma surgeries comprising a significant proportion [6].

Internal fixation (IF) is a surgical technique for stabilizing fractures using plates, screws, nails, or wires and is critical for managing complex fractures and ensuring long-term mobility [7–9]. However, substandard surgical care can lead to infections, disability, or death [10]. A systematic review of 49 studies reported healing rates of 93.7% for femoral shaft fractures and 86.6% for distal fractures, with non-union rates of 2.8% and 4.7%, respectively [7]. Another review found surgical site infections occur in 6.4% of IF cases in LMICs [11]. A multicentre MSF study in Afghanistan and Burundi reported that road traffic accidents (RTAs) accounted for the highest ER presentations (22.9% and 56.4%) compared to violent trauma (11.4% and 8.9%), with IF among the ten most common procedures performed [12]. While IF reduces immobilization time and hospital stays compared to external fixation, it requires specialized resources, trained staff, and rigorous infection control [9, 12].

## Objectives

The primary objective of this study was to evaluate clinical outcomes, including bone union, and postoperative complications among patients undergoing internal fixation surgery at the MSF Aden Trauma Centre in 2022.

## Methods

### Study setting

**Fig. 1:**
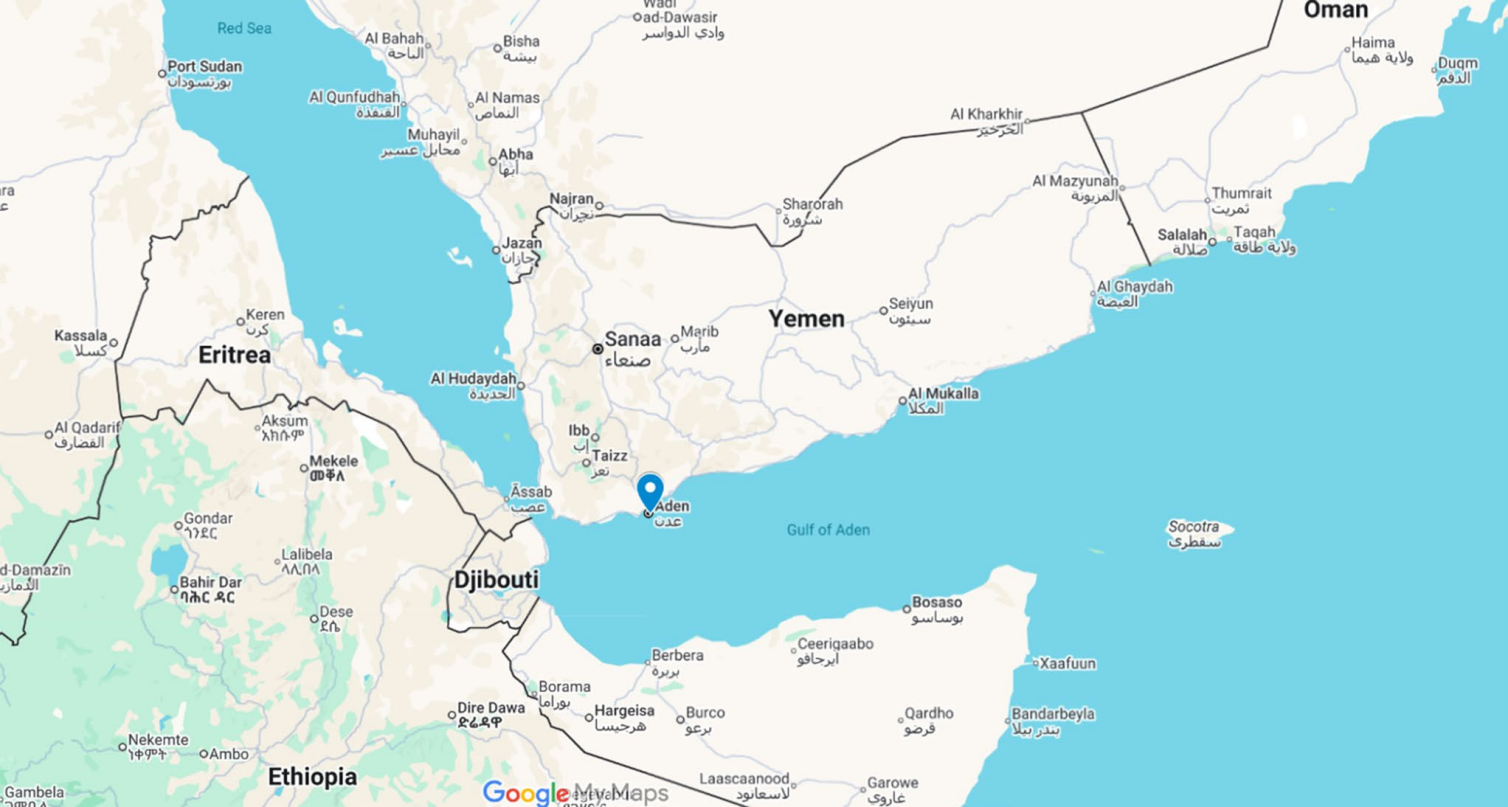
Geographic Location of the MSF Aden Trauma Centre in Yemen

Yemen, located at the southern end of the Arabian Peninsula, has an estimated population of 30 million. Ongoing conflict and socio-economic instability have severely weakened the healthcare system, with only 50% of facilities operational and 20 million people lacking basic healthcare access. Less than 30% of Yemenis can reach emergency surgical care within 30 min [World Bank] (Fig. 1).

 Map showing the location of the Médecins Sans Frontières (MSF) Aden Trauma Centre, where the internal fixation study was conducted in 2022. The pin indicates the study site. Map generated using Google Maps for illustrative purposes.

The Aden Trauma Centre, managed by MSF from 2012 until 2024, provided comprehensive trauma care. It was equipped with a dedicated orthopaedics team, an intensive care unit, a physiotherapy department, an operating theatre for internal fixation (IF), a microbiology laboratory, and an antibiotic stewardship program. Diagnostic services included portable X-ray, CT, and MRI imaging.

### Study design and population

This retrospective cohort study included all patients undergoing internal fixation surgery at the Aden Trauma Centre between January and December 2022, with follow-up extending until August 2023. Routinely collected clinical data were used for analysis.

### Follow-up assessments

Postoperative follow-up visits were conducted over an average period of six months to assess bone healing, functional mobility, and the presence of complications such as infections, non-union, delayed union, and malunion.

### Inclusion and exclusion criteria

Patients who underwent internal fixation procedures for closed or open fractures, according to MSF admission criteria, were included. Exclusion criteria were internal fixation with Kirschner wires for fractures of toes or fingers, tension band wiring for patella fractures, and absence of any postoperative follow-up.

### Outcome variables

The primary outcome was bone healing, categorized as:

- **Union**: Complete healing with radiographic evidence of bridging callus.
- **Non-union or Malunion**: Failure to achieve proper healing or misaligned healing.

Secondary outcomes included postoperative complications (infections, implant failure, readmissions).

### Exposure variables

Exposure variables were categorized into:

- **Demographic variables**: Age, sex, pre-existing comorbidities.
- **Clinical variables**: Fracture type (closed or open) and location (e.g., femur, tibia).
- **Treatment variables**: Type of internal fixation device (SIGN nail or plates/screws).

### Statistical analysis

Descriptive statistics summarized demographics, fracture characteristics, and outcomes. Categorical variables were compared using Chi-square or Fisher’s exact test; continuous variables were analysed using t-tests or ANOVA. A p-value < 0.05 was considered statistically significant.

Time-to-event outcomes, including time to bone union, were analysed using Kaplan-Meier estimates and multivariable Cox proportional hazards models. Analyses were conducted at the procedure level, adjusting for multiple surgeries per patient with robust standard errors clustered by patient ID. A sensitivity analysis to evaluate the robustness of findings was carried out given the number of loss to follow up.

All analyses were performed using RStudio version 2024.04.2 + 764.

### Ethical considerations

The study received ethical exemption from the MSF Ethics Review Board and approval from the local ethics committee.

## Results

### Study flow chart

**Fig. 2:**
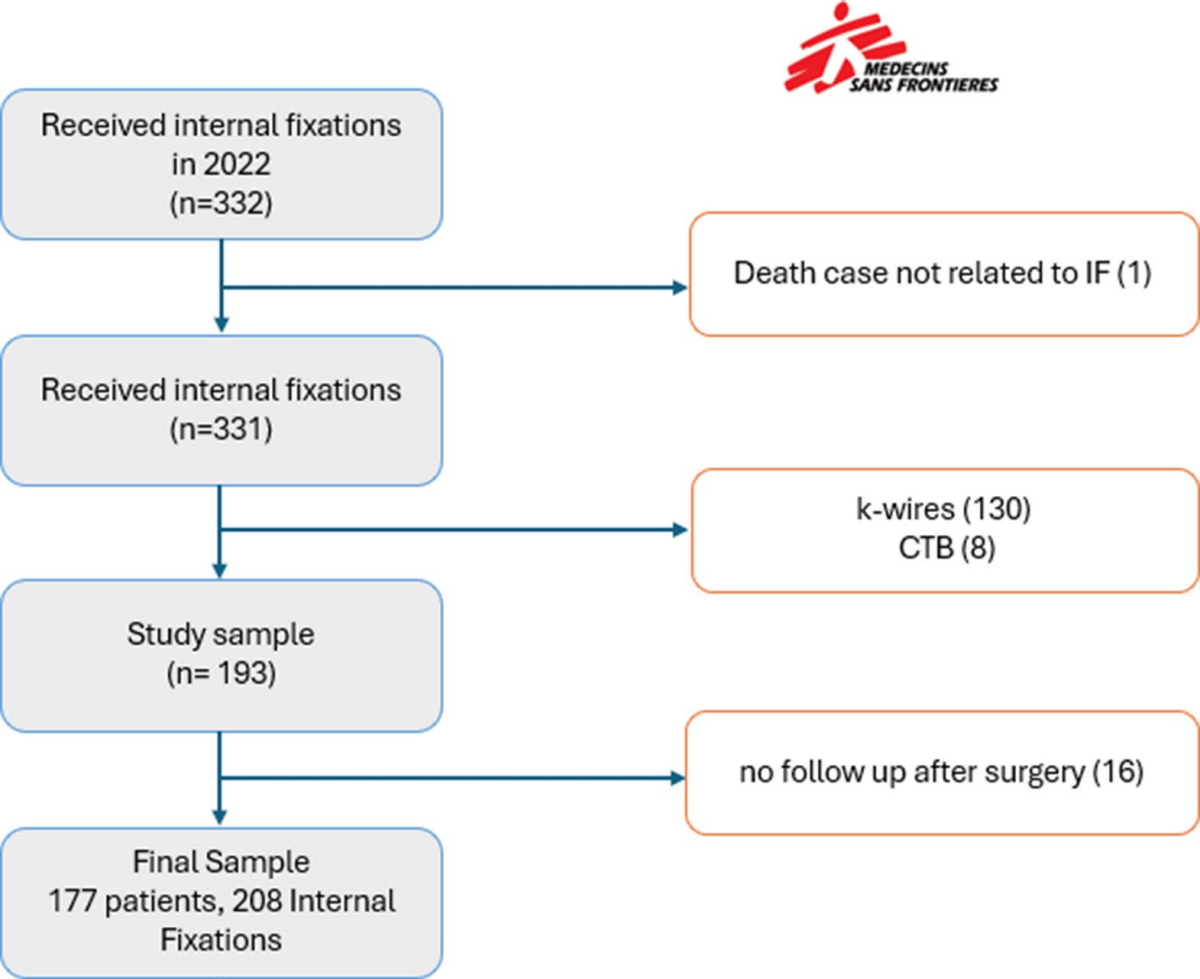
Flow Chart of the study cohort for patients carrying out Internal Fixation Surgery at MSF Aden Trauma Centre, Yemen – 2022

A total of 177 patients met the inclusion criteria, corresponding to 208 internal fixation procedures (Fig. 2).

### Baseline characteristics

The median patient age was 25 years (IQR: 15–43), and 89% were male. Most patients originated from Aden (36%), Lahj (24%), and Abyan (23%) districts. Pre-existing conditions were reported in 9% of patients, predominantly metabolic diseases (Table 1). Road traffic accidents were the leading cause of injury (68%), followed by falls from height (24%). The majority (84%) underwent a single internal fixation procedure.

**Table 1:** Baseline characteristics of patients undergoing internal fixation at the MSF Aden trauma centre in Yemen − 2022 (*N* = 177)

Characteristic	*N* = 177
**Age**,** median (IQR)**	25 (15, 43)
**Sex**,** n (%)**	
Male	157 (89)
Female	20 (11)
**Region**,** n (%)**	
Aden	63 (36)
Lahj	43 (24)
Abyan	40 (23)
Other	31 (18)
**Pre-existing conditions**,** n (%)**	
None	161 (91)
Metabolic Conditions (HBP / Diabetes)	8 (4.5)
Chronic Diseases	8 (4.5)
**Mechanism of Injury**,** n (%)**	
Road Traffic Accident (RTA)	120 (68)
Fall of a height > 3 m	42 (24)
Other traumatic injuries	15 (8.4)
**Number of Internal Fixations per patient**,** n (%)**	
One	148 (84)
More than one	29 (16)

IQR = interquartile range; HBP = high blood pressure

Percentages are column-wise. Data reflect the number of patients, not procedures

### Fracture characteristics by type of internal fixation

Among the 208 fractures, 109 were treated with SIGN nails and 99 with plates and screws (Table 2). All SIGN nail procedures involved lower limb fractures, while plates and screws were used exclusively for upper limb fractures in 54% of cases. Diaphyseal fractures were the most common (82%), particularly among those treated with nails (96% vs. 66%, *p* < 0.001). Plates and screws were more frequently used for distal fractures and simple fractures.

**Table 2:** Fracture characteristics by internal fixation type of patients undergoing internal fixation at the MSF Aden trauma centre, Yemen − 2022 (*N* = 208)

Characteristic	Overall *N* = 208	SIGN nails (*n* = 109)	Plates & Screws (*n* = 99)	*p*-value
**Type of Fracture**,** n (%)**				0.2
Closed	176 (85)	89 (82)	87 (88)	
Open	32 (15)	20 (18)	12 (12)	
**Fracture Location**,** n (%)**				< 0.001
Lower limb	155 (75)	110 (100)	46 (46)	
Upper limb	53 (25)	0 (0)	53 (54)	
**Bone name**,** n (%)**				< 0.001
Femur	108 (52)	86 (79)	22 (22)	
Tibia/Fibula	32 (15)	23 (21)	9 (9.1)	
Radius/Ulna	48 (23)	0 (0)	48 (48)	
Malleolar	15 (7.2)	0 (0)	15 (15)	
Humerus	5 (2.4)	0 (0)	5 (5.1)	
**Fracture Segment**,** n (%)**				< 0.001
Diaphyseal	171 (82)	106 (97)	65 (66)	
Distal	16 (7.7)	0 (0)	16 (16)	
Proximal	6 (2.9)	3 (3)	3 (3)	
Malleolar	15 (7.2)	0 (0)	15 (15)	
**Fracture complexity for femur fractures**				0.036
Simple	89 (52)	47 (44)	42 (65)	
Complex	58 (34)	42 (40)	16 (25)	
wedge	24 (14)	17 (16)	7 (11)	
*Not applicable*	37	3	34	

SIGN = Surgical Implant Generation Network; n = number of procedures

Percentages are based on row totals unless otherwise specified

P-values reflect chi-square or Fisher’s exact tests as appropriate

### Post-operative complications

Complications were recorded in 30 procedures (14.4%) with a median time to event of 40 days (IQR: 16–80). Infection was the most common complication, occurring in 7.7% of procedures overall—5.1% in closed fractures and 22% in open fractures (See Supplementary Table 1). Bone-related complications were reported in 6.7% of procedures.

**Fig. 3:**
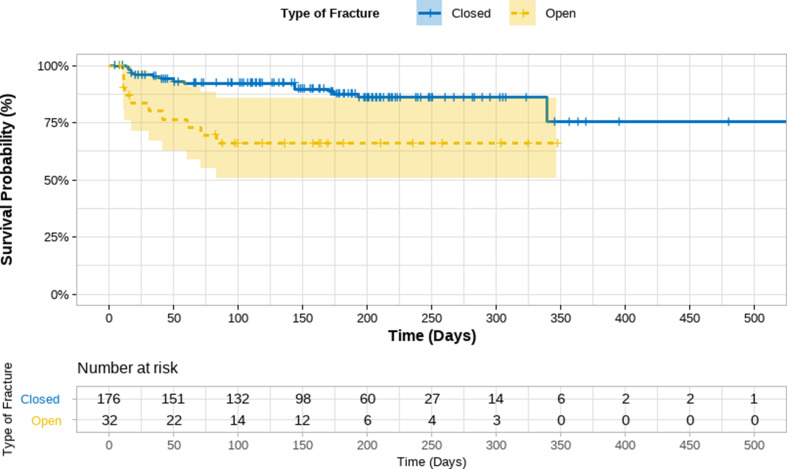
Kaplan Meier Survival Curve for complication free probability by type of fracture of Patients Undergoing Internal Fixation at the MSF Aden Trauma Centre, Yemen − 2022 (*N* = 208)

**Fig. 4:**
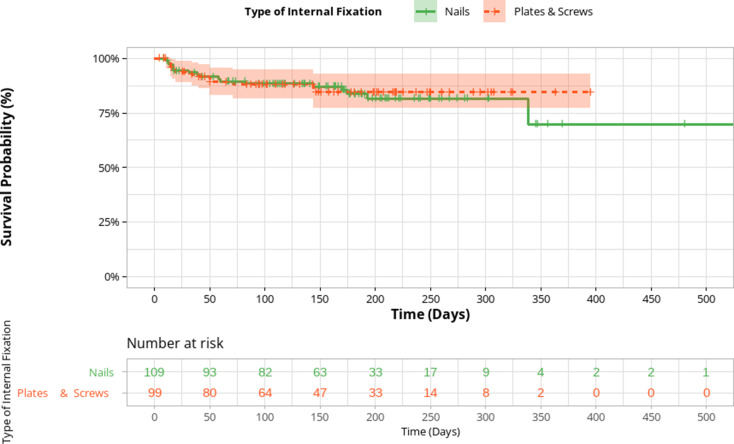
Kaplan Meier Survival Curve for complication free probability by type of Internal fixation surgery at the MSF Aden Trauma Centre, Yemen − 2022 (*N* = 208)

Kaplan-Meier analysis demonstrated a significantly higher complication-free probability among patients with closed fractures compared to open fractures (log-rank *p* < 0.001; Fig. 3). The type of internal fixation was not significantly associated with postoperative complication risk (Fig. 4).

In univariate analysis, children under 15 years had a significantly lower risk of complications (HR 0.19, 95% CI: 0.04–0.79, *p* = 0.023). Open fractures (HR 3.62, 95% CI: 1.69–7.75, *p* < 0.001), the presence of comorbidities (HR 2.57, 95% CI: 1.04–6.32, *p* = 0.04), and other mechanisms of injury (HR 3.69, 95% CI: 1.37–9.95, *p* = 0.01) were associated with a higher risk of complications (Table 3).

**Table 3:** Comparison of patient and fracture characteristics by postoperative complication status following internal fixation at MSF aden trauma centre, Yemen – 2022 (*N* = 208)

Characteristic	With Complication (*N* = 30)	Unadjusted HR (95% CI)	*p*-value
Age Group, *n*(%)			
Adults (> 15 yrs)	28 (93)	Ref	
Children ( = > 15 yrs)	2 (7)	0.19 (0.04–0.79)	0.023
**Sex**,** n(%)**			
Female	3 (10)	Ref	
Male	27 (90)	1.17 (0.35–3.86)	0.8
**Region**,** n(%)**			
Aden	11 (37)	Ref	
Abyan	7 (23)	1.02 (0.39, 2.63)	> 0.9
Lahj	6 (20)	0.82 (0.30–2.23)	0.7
Other	6 (20)	0.98 (0.36–2.68)	> 0.9
**Comorbidities**,** n(%)**			
No	24 (80)	Ref	
Yes	6 (20)	2.57 (1.04, 6.32)	0.04
**Mechanism of Injury**,** n(%)**			
Road Traffic Accident	19 (63)	Ref	
Fall from height	6 (20)	0.90 (0.36–2.24)	0.8
Other	5 (17)	3.69 (1.37, 9.95)	0.01
**Type of fracture**,** n(%)**			
Closed	20 (67)	Ref	
Open	10 (33)	3.62 (1.69, 7.75)	< 0.001
**Fracture Location**,** n(%)**			
Lower Limb	22 (73)	Ref	
Upper limb	8 (27)	1.24 (0.55–2.78)	0.6
**Bone**,** n(%)**			
Femur	14 (47)	Ref	
Humerus	2 (6.7)	4.85 (1.09, 22)	0.038
Malleolar	3 (10)	1.93 (0.55, 6.8)	0.3
Radius/Ulna	6 (20)	1.15 (0.44, 3)	0.8
Tibia/Fibula	5 (17)	1.37 (0.49, 3.84)	0.5
**Fracture segment**,** n (%)**			
Diaphyseal	22 (73)	Ref	
Distal	3 (10)	1.58 (0.47, 5.29)	0.5
Malleolar	3 (10)	1.78 (0.53, 5.96)	0.4
Proximal	2 (6.7)	1.78 (0.40, 7.74)	0.4
**Fracture complexity**,** n(%)**			
Complex	9 (41)	Ref	
Simple	9 (41)	0.59 (0.23, 1.49)	0.3
Wedge	4 (18)	0.93 (0.28, 3.01)	0.9
Not applicable for femur	8		
**Type of Internal fixation**,** n(%)**			
SIGN nails	17 (57)	Ref	
Plates & Screws	13 (43)	0.92 (0.45, 1.90)	0.8

HR = hazard ratio; CI = confidence interval; Ref = reference group

Complication status was assessed at the procedure level P-values from univariate Cox proportional hazards regression.

**Fig. 5:**
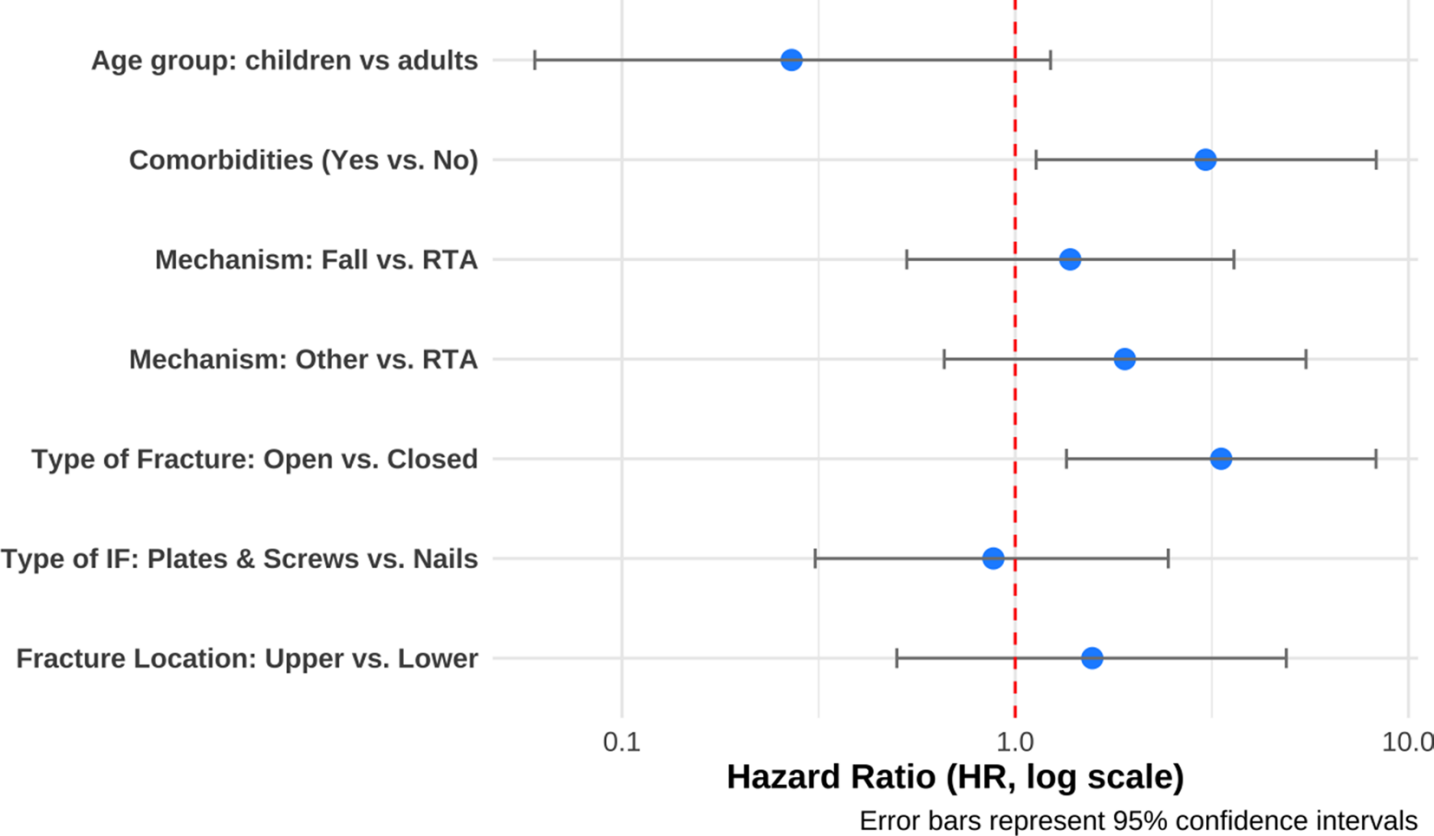
Forest Plot of Multivariate Cox Proportional Hazards Model Showing Risk Factors for Postoperative Complications Following Internal Fixation at MSF Aden Trauma Centre, Yemen – 2022

Multivariable Cox analysis confirmed that open fractures (HR 3.34, 95% CI: 1.35–8.27, *p* = 0.009) and comorbidities (HR 3.5, 95% CI: 1.13–8.28, *p* = 0.028) were independent predictors of complications (Fig. 5).

### Bone union outcomes

By the end of the study period, 72% (*n* = 149) of cases achieved radiological bone union. The median time to union was 162 days (IQR: 116–215). Delayed union occurred in 2.8%, and non-union in 1.4% of cases; 24% of patients were lost to follow-up.

In univariate Cox regression, bone union was more likely among patients from Abyan (HR 1.89, *p* = 0.005) and Lahj (HR 1.65, *p* = 0.018), and among those with radius/ulna fractures (HR 1.57, *p* = 0.024). Complications were significantly associated with a lower likelihood of union (HR 0.26, *p* < 0.001) (Table 4).

**Table 4:** Comparison of patient and fracture characteristics by bone union status following internal fixation at MSF aden trauma centre, Yemen – 2022 (*N* = 208)

Characteristic	Union (*N* = 149)	Unadjusted HR (95% CI)	*p*-value
**Age**,** median (IQR)**	22 (14, 42)	1(0.99, 1.01)	0.8
**Sex**,** n(%)**			
Female	17 (11)	Ref	
Male	132 (89)	0.85 (0.51, 1.41)	0.5
**Region**,** n(%)**			
Aden	54 (36)	Ref	
Abyan	33 (22)	1.89 (1.21, 2.96)	0.005
Lahj	40 (27)	1.65 (1.09, 2.5)	0.018
Other	22 (15)	0.71 (0.43, 1.17)	0.2
**Comorbidities**,** n(%)**			
No	134 (90)	Ref	
Yes	15 (10)	1.26 (0.74, 2.16)	0.4
**Mechanism of Injury**,** n(%)**			
Road Traffic Accident	102 (68)	Ref	
Fall from height	35 (23)	0.89 (0.6, 1.32)	0.6
Other	12 (8.1)	1.72 (0.94, 3.14)	0.076
**Type of fracture**,** n(%)**			
Closed	125 (84)	Ref	
Open	24 (16)	0.93 (0.6, 1.44)	0.7
**Fracture Location**,** n(%)**			
Lower Limb	110 (74)	Ref	
Upper limb	39 (26)	1.41 (0.98, 2.04)	0.068
**Bone**,** n(%)**			
Femur	79 (53)	Ref	
Humerus	1 (0.7)	0.53 (0.07, 3.80)	0.5
Malleolar	8 (5.4)	1.54 (0.74, 3.21)	0.2
Radius/Ulna	38 (26)	1.57 (1.06, 2.32)	0.024
Tibia/Fibula	23 (15)	1.16 (0.73, 1.85)	0.5
**Fracture segment**,** n (%)**			
Diaphyseal	127 (85)	Ref	
Distal	10 (6.7)	1.06 (0.55, 2.02)	0.9
Malleolar	8 (5.4)	1.34 (0.65, 2.75)	0.4
Proximal	4 (2.7)	0.52 (0.19, 1.42)	0.2
**Fracture complexity**,** n(%)**			
Complex	39 (31)	Ref	
Simple	70 (55)	0.84 (0.56, 1.25)	0.4
Wedge	18 (14)	0.64 (0.36, 1.12)	0.12
Not applicable to femur fracture	22		
**Type of Internal fixation**,** n(%)**			
SIGN nails	79 (53)	Ref	
Plates & Screws	70 (47)	1.34 (0.97, 1.87)	0.077
**Any complication**,** n(%)**			
No	140 (94)	Ref	
Yes	9 (6)	0.26 (0.13, 0.51)	< 0.001

HR = hazard ratio; CI = confidence interval; Ref = reference group

Bone union status defined radiologically at final follow-up Union group includes patients with complete healing; p-values from univariate Cox models, Union group includes patients with complete healing; p-values from univariate Cox models

**Fig. 6:**
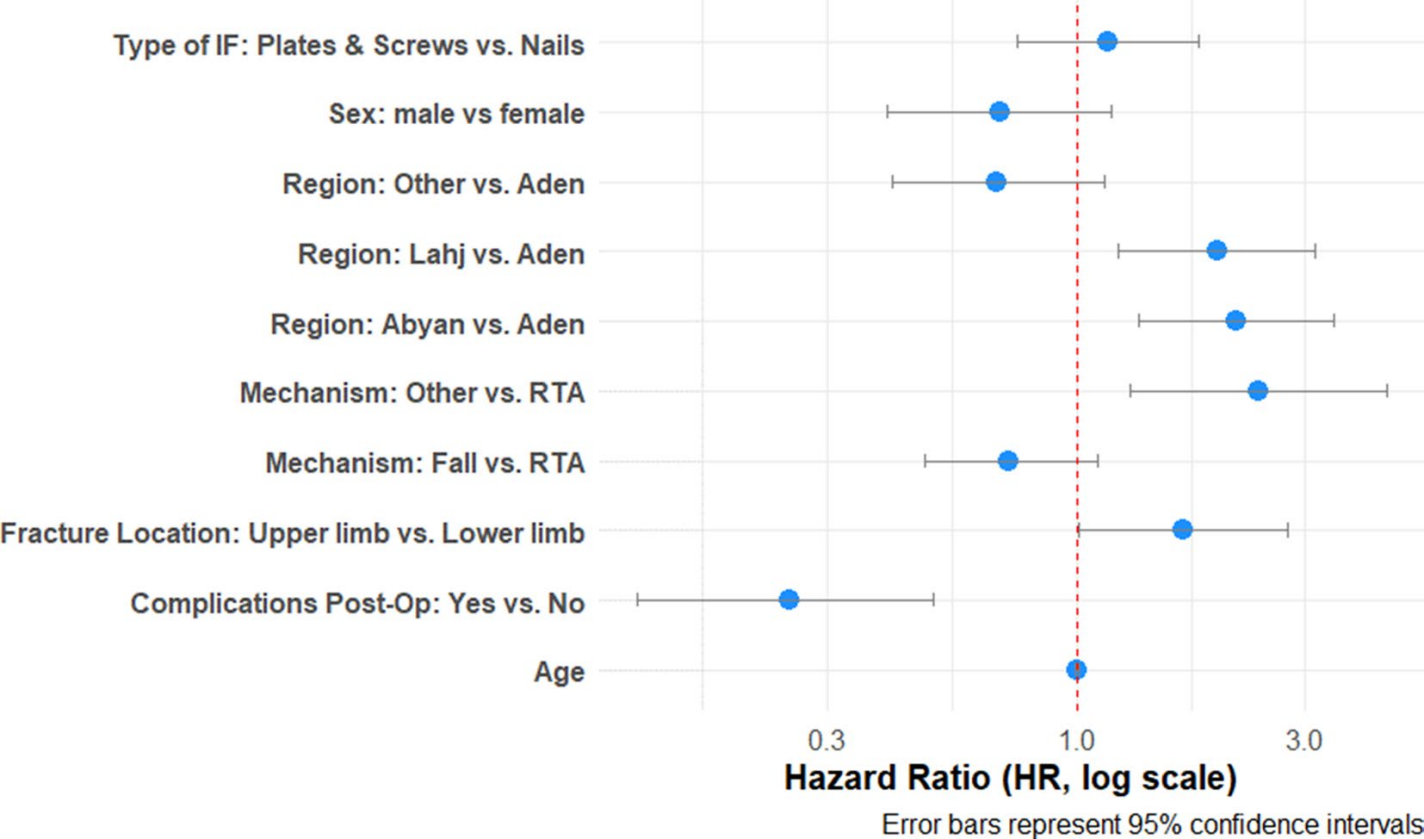
Forest Plot of Multivariate Cox Proportional Hazards Model Showing Risk Factors for Bone Union Following Internal Fixation at MSF Aden Trauma Centre, Yemen – 2022

Multivariable analysis confirmed that patients from Abyan (HR 2.16, *p* = 0.001) and Lahj (HR 1.96, *p* = 0.006), and those with upper limb fractures (HR 1.67, *p* = 0.047), had higher rates of bone union. Conversely, postoperative complications significantly reduced the likelihood of union (HR 0.25, *p* < 0.001) (Fig. 6).

### Loss to follow-up & sensitivity analysis

Loss to follow-up occurred in 24% of cases, with a median time of 65 days (IQR: 21–130) and a median of two visits (IQR: 1–3). Loss to follow-up was more common among patients with humerus and malleolar fractures (*p* = 0.032) and among those who experienced complications (28% vs. 10%, *p* = 0.002) (See Supplementary Table 2).

In sensitivity analysis and if all patients lost to follow-up achieved union at their last recorded visit showed that postoperative complications remained strongly associated with delayed union across models (HR 0.47 vs. 0.25) (See Supplementary Table 3).

## Discussion

Internal fixation (IF) has become increasingly recognized as a viable option for managing bone fractures in low-resource settings, despite concerns around infection risk, implant quality, and surgical capacity limitations. In Aden, MSF has introduced IF procedures to address the high burden of fractures from road traffic accidents (RTAs) and conflict-related injuries. Similar to other low- and middle-income countries (LMICs), barriers such as shortages of trained personnel, inconsistent implant supplies, and limited follow-up capacity persist [9, 13]. However, growing evidence supports that favourable outcomes can be achieved when proper surgical techniques, sterilization, and postoperative protocols are implemented [11, 14, 15]. Our study sought to describe infection rates, bone healing outcomes, and the impact of loss to follow-up in this humanitarian setting.

Postoperative complications occurred in 14.4% of cases, with infections accounting for 7.7%. These rates are consistent with those reported in similar low-resource environments, where infection rates after IF range from 1 to 10%, depending on resource availability and infection control measures [11, 13, 14]. Compared to a recent study assessing the effectiveness of intramedullary nailing for tibial shaft fractures in Yemen, the complications rate was less (18.7%) but with similar infection rate (8.2%) [16]. Open fractures were a major predictor of complications, reflecting findings from other LMIC studies [17]. Comorbidities such as diabetes and hypertension also significantly increased complication risk. Our results showed that implant type (SIGN nail vs. plates/screws) was not associated with complication rates, underscoring the importance of surgical technique, sterilization, and antibiotic prophylaxis over implant selection [7, 14].

Bone union was achieved in approximately 72% of cases, with a median healing time of five to six months, aligning with outcomes reported globally for IF in resource-limited settings. Postoperative complications, particularly infections, were strongly associated with delayed union highlighting the necessity for soft tissue management and infection prevention [11, 13].

Loss to follow-up affected nearly a quarter of patients, a challenge common in LMICs due to geographic, financial, and sociopolitical barriers [12, 18, 19]. Our results also mirrors broader orthopaedic experience in the USA reporting that distance from hospital, lack of insurance and social vulnerabilities are predictors of LTFU [20]. High defaulter rates risk underestimating late complications such as infections and non-union. Similar findings have been reported in the SIGN registry, where improved follow-up revealed higher infection rates [17]. Our sensitivity analysis suggested that the main associations remained robust even when assuming union among lost patients. Nevertheless, improving postoperative follow-up through decentralized care, transport support, or telemedicine could reduce defaulter rates and improve patient management [11]. Another limitation of this study was the lack of patient-level antibiotic data which prevented a thorough analysis. However, all patients received cefazolin prophylaxis preoperatively per MSF protocol. Those with laboratory confirmed infections were treated with intravenous and oral antibiotics following MSF guidelines (Typically 2 weeks IV + 4–6 weeks oral therapy). This limited our ability to directly compare outcomes by antibiotic treatment. Also, future work could adapt low-cost predictive tools, such as Hu et al.’s compartment-pressure nomogram, to identify high-risk patients for intensified follow up and antibiotic stewardship in limited resource settings [21].

## Conclusion

Internal fixation procedures at the Aden Trauma Centre achieved promising outcomes despite resource constraints and challenges with patient follow-up. The modest incidence of complications highlights the effectiveness of targeted measures such as rigorous sterilization, timely prophylactic antibiotics, and surgical training [13, 14]. Infection and open fractures remained critical risk factors for poor outcomes, but overall results support the feasibility of IF in humanitarian contexts when minimum standards are met [7, 11]. Future efforts should focus on strengthening postoperative follow-up and implementing quality improvement initiatives to optimize orthopaedics trauma care in similar settings.

## Electronic supplementary material

Below is the link to the electronic supplementary material.

Supplementary Material 1

## Data Availability

The datasets generated and analyzed during the current study are not publicly available due to confidentiality concerns and operational restrictions but are available from the corresponding author on reasonable request.
