# Supplementary material for: Clinical outcomes of internal fixation orthopaedic surgery in humanitarian settings: a retrospective cohort study at the Médecins Sans Frontières (MSF) trauma centre in Aden, Yemen
Source: Int Orthop. 2025 Aug 13;49(10):2403–13. doi: 10.1007/s00264-025-06616-y (PMC12488807; doi:10.1007/s00264-025-06616-y)
Supplement: Supplementary file 1 — Supplementary Material 1 [file 264_2025_6616_MOESM1_ESM.docx]

**Supplementary Table 1 Complications’ type by Internal Fixation Type of Patients Undergoing Internal Fixation at the MSF Aden Trauma Centre, Yemen - 2022**

| **Complication Type** | **Subcategory** | **SIGN nails** | **Plates & Screws** |
| --- | --- | --- | --- |
| **Operative Complications** | Compartment syndrome | 1 | - |
|  | DVT (Deep Vein Thrombosis) | - | 1 |
|  | Edematous Muscle and Difficulty Performing DPC | - | 2 |
|  | Synovial Fluid Evacuated (Knee Effusion) | 1 | - |
|  | Urinary Catheter-Related Infection with ESBL | 1 | - |
| **Post-operative Bone- Complications** | Poor callus formation | 7 | 4 |
|  | Implant failure (loosening/breaking) | 1 | 2 |
| **Postoperative Infection Complications** | Suspected infection | 1 | 2 |
|  | Deep tissue | 4 | 5 |
|  | Superficial | 2 | 2 |

**Supplementary Table 2 Comparison of Patient and Fracture Characteristics by Loss to Follow-up (LTFU) Status After Internal Fixation at MSF Aden Trauma Centre, Yemen – 2022 (N = 208)**

| **Characteristic** | **No LTFU**  **(N = 158)** | **LTFU**  **(N = 50)** | **p-value** |
| --- | --- | --- | --- |
| **Age, median (IQR)** | 24 (14, 43) | 27 (20, 50) | 0.059 |
| **Sex, n(%)** |  |  |  |
| Female | 18 (11) | 4 (8) | 0.5 |
| Male | 140 (89) | 46 (92) |  |
| **Region, n(%)** |  |  |  |
| Aden | 58 (37) | 19 (38) | 0.12 |
| Abyan | 35 (22) | 12 (24) |  |
| Lahj | 41 (26) | 6 (12) |  |
| Other | 24 (15) | 13 (26) |  |
| **Comorbidities, n(%)** |  |  |  |
| No | 141 (89) | 46 (92) | 0.6 |
| Yes | 17 (11) | 4 (8) |  |
| **Mechanism of Injury, n(%)** |  |  |  |
| Road Traffic Accident | 109 (69) | 32 (64) | 0.8 |
| Fall from height | 37 (23) | 14 (28) |  |
| Other | 12 (7.6) | 4 (8) |  |
| **Type of fracture, n(%)** |  |  |  |
| Closed | 133 (84) | 43 (86) | 0.8 |
| Open | 25 (16) | 7 (14) |  |
| **Fracutre Location, n(%)** |  |  |  |
| Lower Limb | 118 (75) | 37 (74) | >0.9 |
| Upper limb | 40 (25) | 13 (26) |  |
| **Bone, n(%)** |  |  |  |
| Femur | 84 (53) | 24 (48) | 0.032 |
| Humerus | 1 (0.6) | 4 (8) |  |
| Malleolar | 9 (5.7) | 6 (12) |  |
| Radius/Ulna | 39 (25) | 9 (18) |  |
| Tibia/Fibula | 25 (16) | 7 (14) |  |
| **Fracture segment, n (%)** |  |  |  |
| Diaphyseal | 135 (85) | 36 (72) | 0.13 |
| Distal | 10 (6.3) | 6 (12) |  |
| Malleolar | 9 (5.7) | 6 (12) |  |
| Proximal | 4 (2.5) | 2 (4) |  |
| **Fracture complexity, n(%)** |  |  |  |
| Complex | 42 (31) | 16 (44) | 0.3 |
| Simple | 72 (53) | 17 (47) |  |
| Wedge | 21 (16) | 3 (8.3) |  |
| *Not applicable to femur fracture* | *23* |  |  |
| **Type of Internal fixation, n(%)** |  |  |  |
| SIGN nails | 86 (54) | 23 (46) |  |
| Plates & Screws | 72 (46) | 27 (54) |  |
| **Any complication, n(%)** |  |  |  |
| No | 142 (90) | 36 (72) | 0.002 |
| Yes | 16 (10) | 14 (28) |  |

**Supplementary Table 3 Comparison of Adjusted Hazard Ratios for Bone Union: Main Analysis vs. Best-Case Scenario Sensitivity Analysis at MSF Aden Trauma Centre, Yemen – 2022**

| **Characteristic** | **HR (95% CI)**  **– Main Analysis** | **p-value** | **HR (95% CI)**  **– Best-Case Scenario** | **p-value** |
| --- | --- | --- | --- | --- |
| Age | 1.00 (0.99, 1.01) | 0.70 | 1.01 (1.00, 1.02) | 0.11 |
| Sex: Male vs Female | 0.69 (0.41, 1.16) | 0.20 | 0.87 (0.54, 1.38) | 0.50 |
| Region: Abyan vs Aden | 2.16 (1.36, 3.43) | 0.001 | 1.81 (1.23, 2.65) | 0.002 |
| Region: Lahj vs Aden | 1.96 (1.21, 3.17) | 0.006 | 1.41 (0.94, 2.12) | 0.10 |
| Region: Other vs Aden | 0.68 (0.38, 1.23) | 0.20 | 0.88 (0.54, 1.44) | 0.60 |
| Mechanism: Fall vs RTA | 0.72 (0.47, 1.11) | 0.14 | 0.93 (0.64, 1.34) | 0.70 |
| Mechanism: Other vs RTA | 2.40 (1.33, 4.35) | 0.004 | 1.94 (1.14, 3.32) | 0.015 |
| Fracture Location: Upper vs Lower Limb | 1.67 (0.98, 2.84) | 0.059 | 1.24 (0.78, 1.97) | 0.40 |
| Fixation: Plates & Screws vs SIGN nails | 1.16 (0.79, 1.71) | 0.40 | 1.38 (0.96, 1.98) | 0.080 |
| Post-op Complications: Yes vs No | 0.25 (0.11, 0.54) | <0.001 | 0.47 (0.29, 0.78) | 0.004 |
